# The Pathology of Primary Familial Brain Calcification: Implications for Treatment

**DOI:** 10.1007/s12264-022-00980-0

**Published:** 2022-12-05

**Authors:** Xuan Xu, Hao Sun, Junyu Luo, Xuewen Cheng, Wenqi Lv, Wei Luo, Wan-Jin Chen, Zhi-Qi Xiong, Jing-Yu Liu

**Affiliations:** 1 Institute of Neuroscience, State Key Laboratory of Neuroscience, Center for Excellence in Brain Science and Intelligence Technology, Chinese Academy of Sciences, Shanghai, 200031 China; 2 College of Life Science and Technology, Huazhong University of Science and Technology, Wuhan, 430074 China; 3 Department of Neurology and Institute of Neurology of First Affiliated Hospital, Institute of Neuroscience, and Fujian Key Laboratory of Molecular Neurology, Fujian Medical University, Fuzhou, 350005 China; 4 Department of Neurology, The Second Affiliated Hospital, Zhejiang University School of Medicine, Hangzhou, 310009, China

**Keywords:** Primary familial brain calcification, Causative gene, Pathogenesis, Preventive and therapeutic strategy

## Abstract

Primary familial brain calcification (PFBC) is an inherited neurodegenerative disorder mainly characterized by progressive calcium deposition bilaterally in the brain, accompanied by various symptoms, such as dystonia, ataxia, parkinsonism, dementia, depression, headaches, and epilepsy. Currently, the etiology of PFBC is largely unknown, and no specific prevention or treatment is available. During the past 10 years, six causative genes (*SLC20A2*, *PDGFRB*, *PDGFB*, *XPR1*, *MYORG*, and *JAM2*) have been identified in PFBC. In this review, considering mechanistic studies of these genes at the cellular level and in animals, we summarize the pathogenesis and potential preventive and therapeutic strategies for PFBC patients. Our systematic analysis suggests a classification for PFBC genetic etiology based on several characteristics, provides a summary of the known composition of brain calcification, and identifies some potential therapeutic targets for PFBC.

## Introduction

Brain calcification is a common neuropathological phenomenon in the clinic. Its prevalence increases with age (from ~1% in young individuals to 20%–30% in the elderly >60 years) [1–4]. Other than genetic defects, a variety of factors, including endocrine disorders (hypo/hyperparathyroidism and hypothyroidism), intracranial atherosclerosis, infections, brain neoplasms, neurotoxicity, physical injury, and inflammation have been reported to promote or even cause brain calcification [5, 6].

Primary familial brain calcification (PFBC), formerly known as Fahr’s disease or idiopathic basal ganglia calcification, is an inherited and intractable disorder mainly characterized by progressive bilateral calcification distributed in the basal ganglia region and/or other areas of the brain [7]. PFBC can serve as an ideal model in which to study the pathogenesis and potential prevention and treatment of brain calcification. PFBC can result in a variety of clinical symptoms, ranging from occasional migraines to serious symptoms including motor disorders (parkinsonism, tremor, and dystonia), cognitive disorders (memory impairment, executive dysfunction symptoms, and mental retardation), and neurological disorders (depression, affective disorder, and insanity), but nearly one-third of the carriers of causative gene mutation are asymptomatic [3, 8, 9]. Due to the high degree of clinical heterogeneity, the clinical symptoms are not suitable criteria for a diagnosis of PFBC. In contrast, the clinical diagnosis mainly relies on brain calcification identified by computed tomography (CT). The most typical feature in the neuroimages of PFBC patients is symmetrical bilateral calcification in the basal ganglia, thalamus, frontal cortex, or cerebellum. However, the levels of calcium (Ca), phosphorus (P), alkaline phosphatase, parathyroid hormone, and other serum biochemical indicators are normal in PFBC patients [3, 10, 11]. The overall prevalence of PFBC is estimated to be 0.21%–0.66% [12, 13].

It has been >170 years since the initial report of a case of bilateral basal ganglia calcification by Delacour in 1850 [14]. The genetic etiology of PFBC was largely unknown before the identification of *SLC20A2* as the first associated gene in 2012 by Prof. Jing-Yu Liu’s lab [11]. A few pathological studies found that the main component of brain calcification is hydroxyapatite [15, 16]. Calcification particles mainly occur in adventitial vessel cells and sometimes in glial cells, as observed by transmission electron microscopy. Some spherical and hemispherical calcium deposits have been located in the vascular adventitia and connected to the filamentous processes of surrounding cells, as observed by scanning electron microscopy [17]. Furthermore, in some PFBC cases, large spherical calcification particles, mainly calcium phosphate, have been reported to be attached to the capillary wall and located in the media of some large arteries, and reactive astrocytes and microglia have been found to accumulate around the calcification sites [18, 19]. A few neurofibrillary tangles and dystrophic neurites in the medial temporal lobe, rarely spreading senile plaques, and a few amyloid deposits in vascular walls have been detected by silver staining and immunohistochemical staining [20]. Overall, these studies demonstrate that brain calcifications are mostly associated with blood vessels and sometimes involve neurons or glial cells, but the calcification process is still largely unknown.

Since 2012, mutations in 6 genes have been associated with PFBC, including the 4 autosomal dominant genes *SLC20A2*, *PDGFRB*, *PDGFB*, and *XPR1*, and the 2 autosomal recessive genes *MYORG* and *JAM2* [11, 21–25]. Recent cohort studies have expanded the causative gene mutation spectrum by identifying 248 different variants, including 125 in *SLC20A2* (57 missense, 15 nonsense, 11 splicings, 30 small deletions, 4 small insertions, 1 intronic, and 7 gross deletions), 15 in *PDGFRB* (14 missense and 1 start loss), 26 in *PDGFB* (9 missense, 4 nonsense, 5 splicings, 1 small deletion, 1 small insertion, 2 start loss, 2 stop loss, 1 gross deletion, and 1 complete deletion), 14 in *XPR1* (13 missense and 1 small insertion), 59 in *MYORG* (34 missense, 9 nonsense, 9 small deletions, 6 small insertions, and 1 small indel), and 8 in *JAM2* (2 missense, 1 nonsense, 1 splicing, 2 small deletions, 1 start loss, and 1 gross deletion). The frequencies of these genes counted from 555 individuals with PFBC were 59.78%, 5.98%, 12.68%, 5.98%, 13.59%, and 1.99%, respectively [9, 26–39] (http://www.hgmd.cf.ac.uk/ac/index.php), but the spectrum may vary among different cohorts and countries. Some mechanistic studies of these causative genes at the cellular level and in animals have been reported, contributing to the understanding of PFBC pathogenesis.

In this review, we focus on studies of the biological functions of the causative genes *SLC20A2*, *PDGFRB*, *PDGFB*, *XPR1*, *MYORG*, and *JAM2* to summarize PFBC pathogenesis and potential strategies for PFBC prevention and treatment.

## The Genetic Etiology of PFBC

Based on the functions of the reported causative genes (*SLC20A2*, *PDGFRB*, *PDGFB*, *XPR1*, *MYORG*, and *JAM2*), the genetic etiology of PFBC can be classified into two categories: imbalance of inorganic phosphate (Pi) and dysfunction of the neurovascular unit (NVU) in the brain. The Pi levels in the cerebrospinal fluid (CSF) from PFBC patients are known to be an overall increase compared with controls [40], implying that impairment of cerebral Pi homeostasis is a major factor in brain calcification. Mutations of *SLC20A2* and *XPR1* resulting in cellular Pi imbalance can be direct contributors to the increased CSF Pi levels, and mutations of *PDGFRB*, *PDGFB*, *MYORG*, and *JAM2* leading to NVU dysfunction may be indirect contributors to the increased CSF Pi levels.

### Pi Imbalance

In the brain, Pi homeostasis is closely related to changes in Pi levels in CSF, brain interstitial fluid (ISF), cerebral blood, and intracellular fluid, the regulation of which is mainly dependent on Pi transporters. Since CSF occurs in the ventricles and subarachnoid space, it is easier to acquire and test than other cerebral fluids, and therefore is the currently best choice to reflect changes in Pi levels in the brain. In both blood and CSF, there is a dynamic shuttle of phosphate between its free inorganic form and various Pi-containing compounds, which results in different outcomes in the measurement of inorganic Pi concentrations by different kits from different suppliers. Using a clinical spectrophotometry method and a malachite green and ammonium molybdate-based colorimetric approach, the concentration of CSF Pi is reported to be maintained at ~0.6 mmol/L in humans and ~0.9 mmol/L in mice [41, 42].

#### SLC20A2

*SLC20A2* encodes type III sodium-dependent phosphate (Na^+^/Pi) transporter 2 (PiT2), which was originally identified as a receptor for amphotropic murine retroviruses and soon shown to be able to transport extracellular Pi into the cell depending on excess sodium ions with a 2:1 Na^+^:Pi transport stoichiometry [43–45]. *In vitro*, *SLC20A2* mutations impair the cellular Pi transport activity of PiT2, leading to the accumulation of extracellular Pi [11, 46], indicating that the formation of brain calcification may be associated with cerebral Pi dyshomeostasis. Recently, changes in PiT2 expression levels have been shown to affect not only Pi uptake but also some Pi efflux [47]. In animal models, *Drosophila* cannot be used for mechanistic studies of PFBC due to embryonic lethality resulting from a deficiency of dPiT (the protein homologous to human PiT2) [48]. Mice with PiT2 deficiency develop widespread brain calcification and exhibit abnormal multisystem phenotypes, including placental calcification, fetal growth restriction, developmental delay, lean body mass, lower bone quality and strength, skeletal malformation, a high likelihood of eye defects, impaired spatial learning, memory, and sensorimotor gating [49–52].

**Fig. 1:**
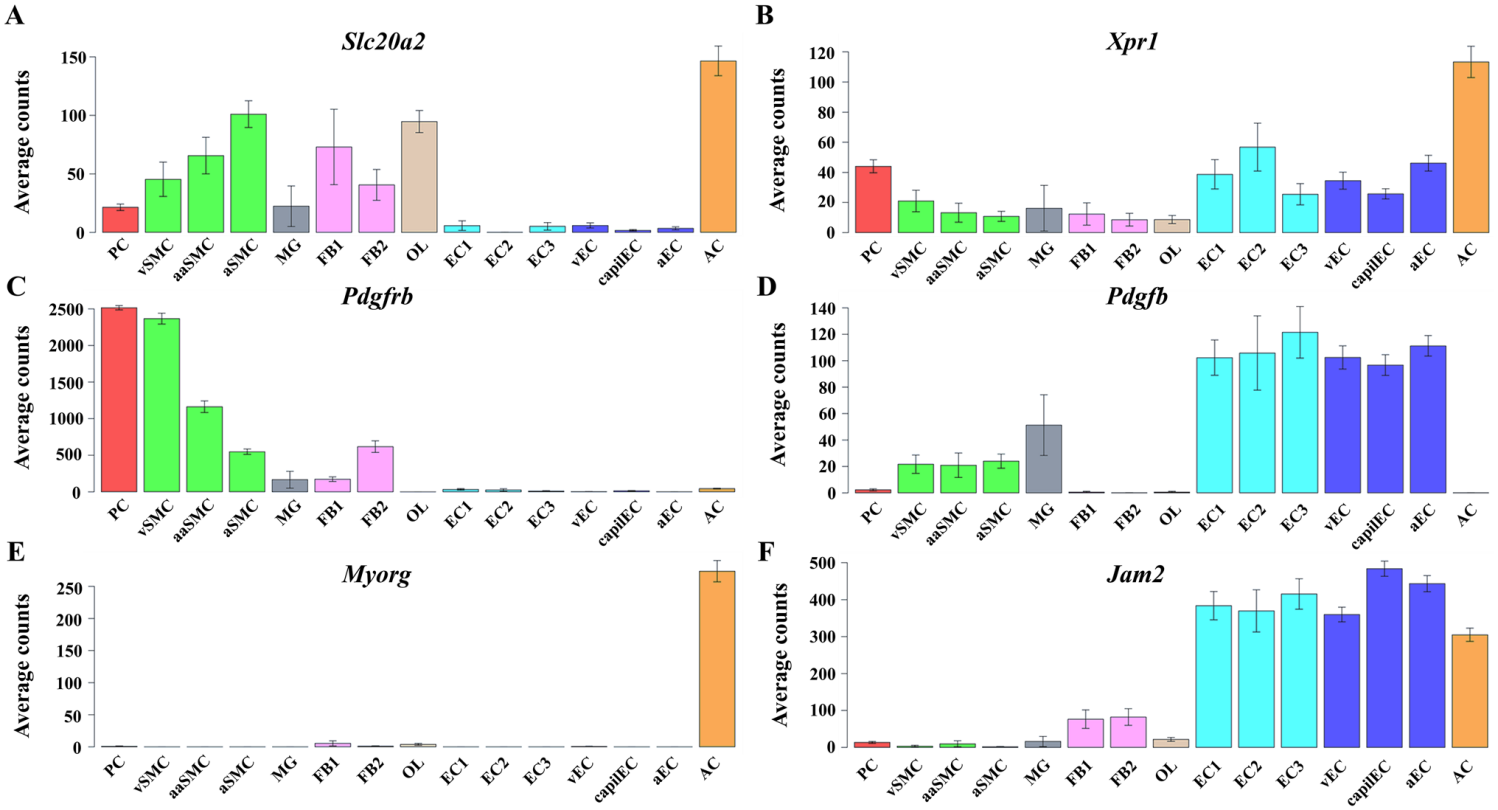
Expression of genes in mouse NVU-related cells [56] (https://www.proteinatlas.org/). **A–F** Single cell RNA-seq of the mouse brain database showing the expression of (**A**) *Slc20a2*, (**B**) *Xpr1*, (**C**) *Pdgfrb*, (**D**) *Pdgfb*, (**E**) *Myorg*, and (**F**) *Jam2* in NVU-related cells. PC, pericytes; vSMC, venous smooth muscle cells; aaSMC, arteriolar smooth muscle cells; aSMC, arterial smooth muscle cells; MG, microglia; FB1, type 1 vascular fibroblast-like cells; FB2, type 2 vascular fibroblast-like cells; OL, oligodendrocytes; EC1, type 1 endothelial cells; EC2, type 2 endothelial cells; EC3, type 3 endothelial cells; vEC, venous endothelial cells; capilEC, capillary endothelial cells; aEC, arterial endothelial cells; AC, astrocytes.

Furthermore, compared with wild-type mice, homozygous *Slc20a2*-knockout (*Slc20a2*^−/−^) mice display a dramatically elevated Pi level in CSF [42, 53], consistent with the increase in CSF Pi level in PFBC patients with *SLC20A2* mutations [40]. Considering that CSF flowing along the paravascular spaces exchanges metabolites with parenchyma ISF [54, 55], it is reasonable to expect that the ISF Pi level also undergoes an increase similar to CSF Pi. In addition, PiT2 is widely and strongly expressed in neurons, astrocytes, oligodendrocytes, microglia, vascular smooth muscle cells (VSMCs), and vascular fibroblast-like cells (Fig. 1) [56] (https://www.proteinatlas.org/), and deficiencies or mutations of PiT2 in these cells probably have impaired Pi transport activity leading to the accumulation of extracellular ISF Pi. Moreover, PiT2-deficient VSMCs have been shown to present high Pi-induced calcification and elevated expression of Runx2 and osteopontin (OPN) [57]. Thus, the high extracellular Pi environment may induce intracranial VSMC calcification, possibly *via* transdifferentiation of VSMCs into osteoblast-like cells, an initial phase similar to the mineralization process during bone development [57–59].

How do Pi levels increase in CSF in response to PiT2 deficiency or *SLC20A2* mutations? CSF is predominantly secreted by the choroid plexus in the lateral, third, and fourth ventricles, and may also be associated with some poorly-defined sources such as ISF, ependyma, and capillaries [60–62]. In the brain, type III transporters (PiT1 and PiT2) are the major detectable Na^+^/Pi transporters [63, 64], which are highly tissue-specifically expressed in the choroid plexus. PiT1 is largely expressed in the vascular endothelium of the choroid plexus facing the blood, while PiT2 is mainly expressed in the apical membrane region facing the CSF [53, 63], implying that PiT1 contributes to the transport of Pi from blood to CSF and PiT2 contributes to the transport of Pi from CSF to blood. A single impairment of PiT2 may largely reduce Pi transportability from CSF to blood, which leads to Pi accumulation in the CSF. On the other hand, the likely increased ISF Pi level may also contribute to the increased CSF Pi level *via* substance exchange in the CSF-ISF system.

**Fig. 2:**
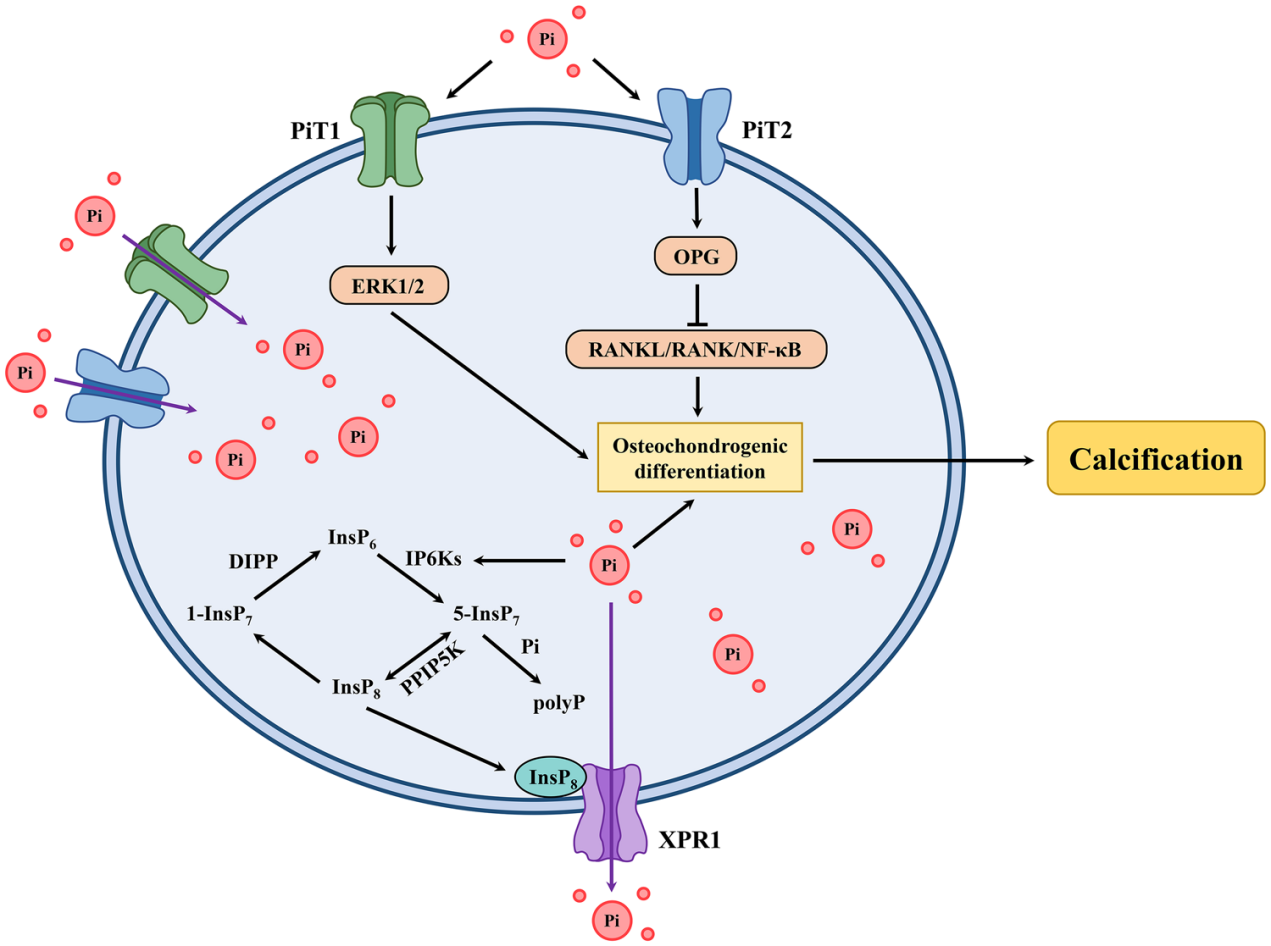
Potential mechanism of Pi transport and calcification regulated by Pi transporters at the cellular level. Intracellular Pi levels are in a dynamic balance regulated by the Pi importers PiT2/PiT1 and the Pi exporter XPR1. Under physiological conditions, PiT2 inhibits osteochondrogenic differentiation and calcification by activating OPG (osteoprotegerin) and inhibiting NF-κB/RANKL/RANK signaling. However, increased extracellular Pi can induce osteochondrogenic differentiation and calcification by activating PiT1/ERK1/2 signaling. XPR1-dependent Pi efflux is regulated by XPR1/InsP_8_ signaling, deficiency of which increases intracellular Pi and induces osteochondrogenic differentiation and calcification. Excessive intracellular Pi can be stored by the synthesis of polyP *via* IP6Ks and 5-InsP_7_. ERK1/2, extracellular regulated protein kinases 1/2; DIPP, diphosphoinositol polyphosphate phosphohydrolase; InsP_8_, 1,5-bis-diphosphoinositol 2,3,4,6-tetrakisphosphate; PPIP5K, diphosphoinositol pentakisphosphate kinase; IP6Ks, inositol hexakisphosphate kinases; 5-InsP_7_, 5-diphosphoinositol 1,2,3,4,6-pentakisphosphate; 1-InsP_7_, 1-diphosphoinositol 1,2,3,4,6-pentakisphosphate; InsP_6_, inositol hexakisphosphate; polyP, intracellular inorganic polyphosphate.

Mechanistically, at the plasma membrane, PiT2 and PiT1 are mostly in the form of homodimers, but some are in the form of Pi-regulated heterodimers, which may mediate extracellular Pi signaling [65]. Under high extracellular Pi conditions, PiT2 deficiency increases VSMC calcification by reducing the expression of osteoprotegerin (OPG), supplementation of which can attenuate calcification [57]. OPG is an anti-calcification factor that inhibits NF-κB/RANKL/RANK (nuclear factor kappa B/receptor activator of NF-κB ligand/receptor activator of NF-κB) signaling, which plays important roles in Pi-induced osteochondrogenic differentiation and vascular calcification [66, 67]. In contrast, PiT1 plays a role in promoting high Pi-induced osteochondrogenic differentiation and VSMC calcification *via* extracellular regulated protein kinases 1/2 (ERK1/2) phosphorylation independent of Pi uptake. Knockdown of PiT1 abrogates high Pi-induced ERK1/2 signaling, which is rescued by supplementation with transport-deficient PiT1 mutants or wild-type PiT1 (Fig. 2) [68, 69].

#### XPR1

*XPR1* encodes xenotropic and polytropic murine virus receptor 1 (XPR1), which was originally identified as a retroviral receptor [70, 71], and later shown to be able to export intracellular Pi out of the cell; XPR1 is the only known Pi exporter in metazoans [72]. The amino terminus of XPR1 contains an SPX (named after the proteins SYG1, PHO81, and XPR1) domain that was recently identified as an intracellular sensor of Pi level and homeostasis in both plants and animals [73, 74]. *In vitro* studies found that mutations in the SPX domain of XPR1 or short interfering RNA-mediated knockdown of XPR1 lead to decreased Pi export [23], suggesting that missense mutations in *XPR1* in PFBC patients may also be associated with impaired brain Pi homeostasis. Recently, knockout of XPR1 in human cell lines was reported to cause not only a significant reduction in Pi efflux but also to markedly down-regulate Pi uptake, suggesting a potential synergistic mechanism to coordinate Pi uptake and export. Moreover, when incubated in a high Pi medium, cultured human Saos-2 cells with *XPR1* deficiency show increased expression of osteocalcin, osteonectin, and alkaline phosphatase, and finally accumulation of calcification deposits [75].

In animal models, *xpr1b* (the orthologue of human *XPR1* in zebrafish) mutations in zebrafish lead to a lack of microglia in the brain and an osteopetrotic phenotype, but no data on brain calcification have been reported [76]. Heterozygous *Xpr1*-knockout mice older than 1 year fail to show a brain calcification phenotype (unpublished data), and the homozygotes die perinatally [77]. Complete knockout of *Xpr1* seems to not be a good choice for generating a PFBC model, but knock-in or conditional knockout of *Xpr1* in cerebral cells such as neurons and astrocytes may be worth attempting. In other tissue studies associated with Pi transport, XPR1 deficiency in mice results in severe placental calcification, reduced placental Pi exchange, lower Pi levels in amniotic fluid and serum, and decreased fetal skeletal mineral content [77]. Conditional inactivation of *Xpr1* in the renal tubule of mice impairs renal Pi reabsorption, which is accompanied by glycosuria, aminoaciduria, calciuria, albuminuria, hypophosphatemic rickets, reduction in NaPi-IIa/NaPi-IIc expression, and vertebral osteomalacia [78]. These results indicate that XPR1 deficiency impairs tissue Pi homeostasis and may be an inducible factor of ectopic calcification.

In the brain, the only known Pi exporter, XPR1, is widely expressed in various cerebral cells and is particularly highly expressed in neurons, astrocytes, and microglia (Fig. 1) [56] (https://www.proteinatlas.org/). It must be expected that mutations in *XPR1* impair Pi balance in cerebral cells, but the detailed effects of Pi levels in CSF and ISF are largely unknown. In addition to the lack of direct measurements of CSF and ISF Pi levels, studies of the tissue-specific expression of XPR1 in the structures of substance exchange systems, such as the choroid plexus of the blood-CSF barrier (BCB) and blood-brain barrier (BBB), are lacking.

How does XPR1 regulate Pi homeostasis and calcification at the cellular level? Recent studies have shown that XPR1-dependent Pi efflux is specifically regulated by a member of the inositol pyrophosphate (PP-InsP) signaling family, 1,5-bis-diphosphoinositol 2,3,4,6-tetrakisphosphate (InsP_8_), which binds to the SPX domain of XPR1 [75]. The multiple upstream pathways of InsP_8_, including diphosphoinositol pentakisphosphate kinases (PPIP5Ks), 5-diphosphoinositol 1,2,3,4,6-pentakisphosphate (5-InsP_7_), inositol hexakisphosphate kinases (IP6Ks), and inositol hexakisphosphate (InsP_6_), indirectly affect XPR1-dependent Pi efflux [47, 75, 79]. Deficiency of XPR1/InsP_8_ signaling results in an elevated intracellular Pi environment, which contributes to the induction of an osteoblastic phenotype and calcification in Saos-2 cells [75]. In addition, inhibition of XPR1 leads to the accumulation of intracellular inorganic polyphosphate (polyP) [80], a major Pi storage molecule, the synthesis of which is also associated with IP6Ks and 5-InsP_7_ (Fig. 2) [81–84].

### NVU Dysfunction

**Fig. 3:**
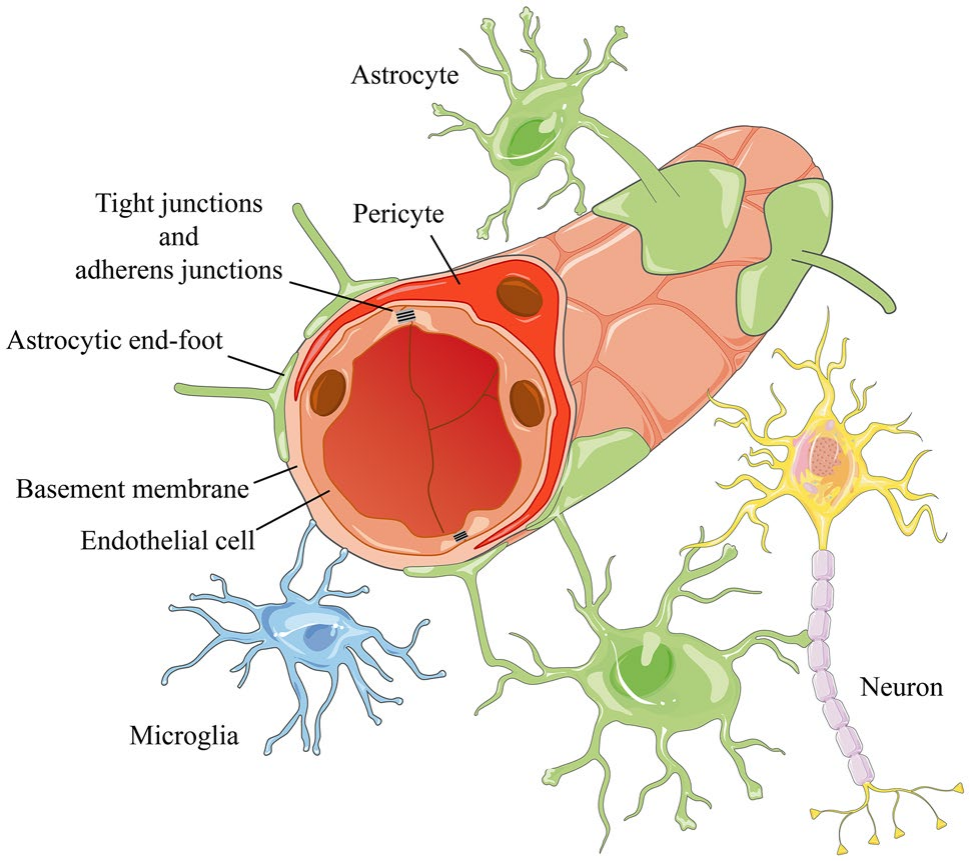
Structural diagram of an NVU (neurovascular unit). Endothelial cells form the tubular structure of the cerebral capillaries *via* the connection of tight junctions and adherens junctions. The basement membrane is embedded between endothelial cells and pericytes. Astrocytic end-foot processes ensheath the vascular wall. Microglia and neurons surround the vascular wall.

The concept of the NVU was first described in 2001 to emphasize the unique relationship between brain cells and cerebral blood vessels (https://www.ninds.nih.gov/About-NINDS/Strategic-Plans-Evaluations/Strategic-Plans/Stroke-Progress-Review-Group). The NVU comprises neurons, glial cells (including astrocytes, microglia, and oligodendroglia), and vascular cells (including endothelial cells, pericytes, and VSMCs) [85, 86]. Specifically, the tubular structure of the cerebral capillaries is composed of endothelial cells. Adjacent endothelial cells are connected by tight junctions and adherens junctions. Tight junctions are mainly composed of occludin, claudins, and junctional adhesion molecules (JAMs). The outer edges of endothelial tubes are surrounded by pericytes and astrocytic end-feet (Fig. 3) [85, 87, 88]. The BBB is centrally located within the NVU and consists of a continuous endothelial cell membrane structure and coverage structures of endothelial cells by the basement membrane and pericytes [89–91].

An NVU, as the minimal functional unit in the brain, plays a vital role in regulating cerebral blood flow and maintaining BBB integrity, dysfunction of which impairs the normal substance exchange between blood and cerebral parenchyma. In humans, the Pi level in CSF (~0.6 mmol/L) is significantly lower than that in serum (~1.1 mmol/L) [41]. If the BBB is impaired, the increased BBB permeability probably leads to a high concentration of blood Pi leaking into the brain.

#### PDGFRB and PDGFB

*PDGFRB* encodes platelet-derived growth factor receptor beta (PDGFR-β), and its ligand protein, platelet-derived growth factor B (PDGF-B), is encoded by *PDGFB* [89, 92–94]. PDGFR-β is a transmembrane tyrosine kinase receptor for platelet-derived growth factor family members (PDGF-A, B, C, and D). In the mammalian brain, PDGFR-β is expressed mainly by pericytes, VSMCs, and vascular fibroblast-like cells [92, 95, 96]. On the contrary, PDGFB is predominantly expressed by vascular endothelial cells (vECs) and neurons (Fig. 1) [56]. As a paracrine factor, secreted PDGF-B homodimer enables vECs to recruit chemotactic PDGFR-β-expressing pericytes and VSMCs, thereby promoting the envelopment or wrapping of the cerebral vessel network by pericytes and VSMCs to consolidate the cellular basis of BBB integrity [97, 98]. Deficiency of PDGF-B/PDGFR-β signaling leads to microvascular pericyte deficiency, endothelial hyperplasia, increased vessel diameter, abnormal endothelial cell shape and ultrastructure, the abnormal cellular distribution of junctional proteins (occludin and vascular endothelial-cadherin), and morphological signs of increased vascular permeability [92, 93]. Adult mice with partial inactivation of *Pdgfb* or *Pdgfrb* also present with defects in pericyte generation, which impairs BBB integrity and increases BBB permeability due to the activation of endothelial transcytosis and astrocytic end-foot polarization [89, 91].

*In vitro*, missense mutations in PDGFR-β interfere with the activation of PDGFR-β and its downstream effectors *via* reduced autophosphorylation or protein levels [99]. Similarly, several functional studies subsequently demonstrated that PDGFR-β autophosphorylation, in response to PDGF-BB (the dimer of PDGF-B) stimulation, is abolished/reduced due to PDGFB or PDGFRB mutations [99–101], indicating that a functional loss of PDGF-B/PDGFR-β may be the cause of PFBC. Interestingly, several studies have reported that PDGF-BB induces calcification in VSMC lines accompanied by increased expression of PiT1 in the endoplasmic reticulum and an increased Pi transport rate [102, 103]. Based on these findings, Nicolas *et al*. put forward the opposite hypothesis that an activating mutation in PDGF-B/PDGFR-β may lead to brain calcification due to alterations in the PDGF-PiT1 pathway directly inducing vascular calcification [22]. However, no further evidence has supported this hypothesis. Recently, PDGF-BB was shown to increase intracellular Pi uptake by regulating the membrane migration of PiT-1 by activating protein kinase B (AKT) signaling in human neuroblastoma SH-SY5Y cells [104], indicating that increased PDGF-B/PDGFR-β signaling may contribute to intracellular Pi uptake in the central nervous system (CNS), but it is unclear whether decreased PDGF-B/PDGFR-β signaling in the CNS has an influence similar to *SLC20A2* mutations on normal intracellular Pi uptake and leads to extracellular Pi accumulation.

As both homozygous *Pdgfb*- and *Pdgfrb*-knockout mice die perinatally [105, 106], these mouse lines are not good models in which to simulate brain calcification. However, the mutant line *Pdgfb*^ret/ret^, with a disrupted PDGF-B retention motif that impairs its maturation and binding to the membrane [107], develops progressive calcification in the brain [21]. Another mouse line, *Pdgfb*^−/−^; *R26P*^+/0^ mice, with a 50% level of rescue by transgenic re-expression of human PDGF-B in the endothelium [89], develop smaller and fewer lesions but with a similar histological appearance and anatomical location [21], suggesting a strong correlation of endothelial PDGFB deficiency with brain calcification. Pathologic studies have shown that with respect to the levels in control mice, *Pdgfb*^ret/ret^ mice display a more profound reduction in pericyte coverage and a greater increase in BBB permeability than *Pdgfb*^+/−^; *Pdgfrb*^+/−^ or *Pdgfrb*^redeye/redeye^ mice [101]. These results imply that brain calcification resulting from a reduction in PDGF-B/PDGFR-β signaling may be associated with the degree of pericyte loss and/or BBB impairment, which contributes to the high concentration of blood Pi leaking into the brain. In a neuropathological study of one PFBC case, impaired BBB integrity with substance leakage from blood to the brain was identified in calcified brain areas [18], which supports the above speculation. However, the possibility that the integrity of the BBB in this case was destroyed by increased calcification cannot be excluded. Another study showed that PFBC patients carrying *PDGFB* mutants display a slight increase in the CSF Pi level, further supporting the aforementioned phenomena and speculation [40].

#### MYORG

*MYORG* encodes a putative myogenesis-regulating glycosidase (MYORG), which contains an N-terminal transmembrane domain and a C-terminal predictive glycosidase domain. It is widely expressed in many tissues [108], but its molecular function remains largely unknown. The homozygous nonsense mutations in PFBC patients suggest a causal association of the functional loss of *MYORG* with brain calcification [24]. In animal models, homozygous *Myorg*-knockout (*Myorg*^−/−^) mice are viable and display calcification in the thalamus at 9 months of age, which is much later than that reported in *Slc20a2*^−/−^ and *Pdgfb*^ret/ret^ mice. In contrast to the enriched expression in perivascular cell types by other causative genes, MYORG has been found to be predominantly expressed in astrocytes (Fig. 1) [56], and is mainly localized to the endoplasmic reticulum [24]. Astrocytes function as essential metabolic intermediates between neuron-centralized intra-parenchyma circumstances and NVU-connected blood circulation, the dysfunction of which is frequently found to cause abnormal BBB function and neuroinflammation in neurodegenerative diseases [109]. Thus, it is interesting to explore whether the function of astrocytes is impaired in both *Myorg*-deficient mice and human patients, which in turn might result in the disturbance of NVU permeability, or otherwise decoy causative changes of surrounding pericytes and smooth muscle cells, paving the way for calcification development.

#### JAM2

*JAM2* encodes junctional adhesion molecule 2 (JAM2), a member of the JAM family, and is a key component of the tight junctions between adjacent endothelial cells in the NVU [25]. In the brain, JAM2 is expressed mainly in endothelial cells and astrocytes and to a lesser degree in vascular fibroblast-like cells (Fig. 1) [56]. Functional studies of *JAM2* variants have demonstrated that *JAM2*-associated PFBC results from the effects of JAM2 loss-of-function [25, 110]. Homozygous *Jam2*-knockout (*Jam2*^−/−^) mice present with no calcification but prominent vacuolation with reactive astrogliosis and neuronal density reduction in the brain at the age of 6 or 18 months. Interestingly, in the spinal cord of *Jam2*^−/−^ mice, in addition to vacuolation, mineralized deposits are widely evident in the grey matter [110]. In addition to mutations in JAM2, biallelic mutations in the tight junction proteins occludin and JAM3 (a member of the JAM family and a counter-receptor of JAM2 [111]) have also been reported to result in brain calcification in congenital syndromes in humans [112, 113]. JAM3 mutations or deficiencies in humans and mice also result in hemorrhages in the brain and hydrocephalus [114, 115], suggesting that JAM3 is associated with the regulation of BBB integrity. Taken together, we speculate that *JAM2* mutants probably impair the tight junctions between adjacent endothelial cells in the BBB and/or BCB, leading to increased BBB and/or BCB permeability with high Pi leakage from the blood to the brain, which contributes to an increased Pi level in the CSF.

In addition to regulating the formation of tight junctions, JAMs participate in the immune cell response by regulating the transendothelial migration of leukocytes [116, 117], but this has not yet been linked to calcification. Much more research on the relationship between the functional duality of JAMs and brain calcification is needed.

**Fig. 4:**
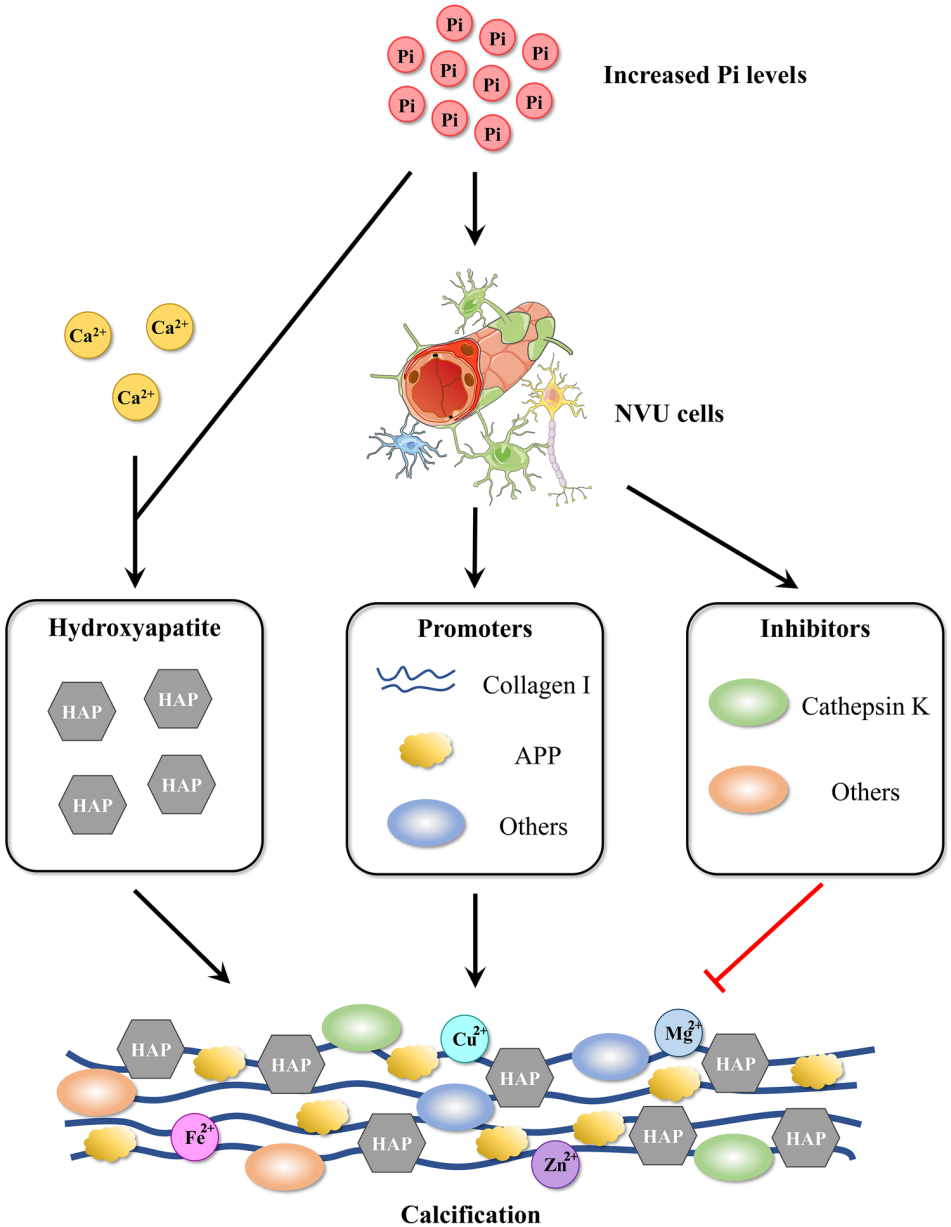
Putative mechanism of brain calcification induced by increased Pi levels. In the brain, increased Pi levels may induce the ossification of NVU cells contributing to the production of organic substances for calcification. On the other hand, increased Pi levels may contribute to combination with Ca^2+^ to form hydroxyapatite. After a series of combinations of organic substances and inorganic substances including hydroxyapatite, Cu^2+^, Zn^2+^, Fe^2+^, and Mg^2+^, calcified crystals form and grow larger. HAP, hydroxyapatite; APP, amyloid precursor protein.

**Fig. 5:**
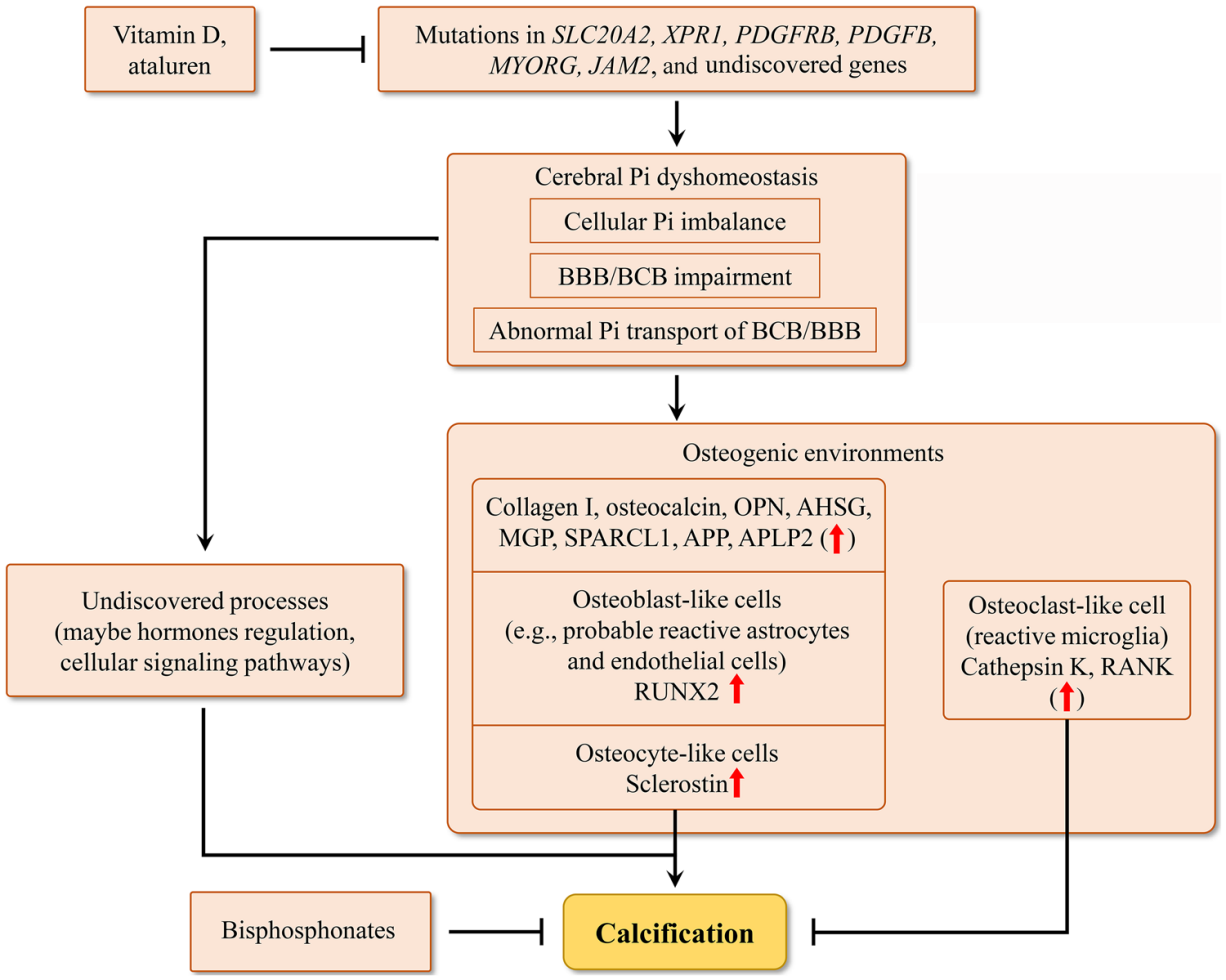
Structural diagram of the potential pathogenesis and potential prevention/treatment of PFBC. BBB, blood-brain barrier; BCB, blood-CSF barrier; OPN, osteopontin; AHSG, fetuin A; MGP, matrix gla protein; SPARCL1, an ancestral mineralization protein sparc-like 1; APP, amyloid precursor protein; APLP2, amyloid precursor-like protein 2; RUNX2, runt-related transcription factor-2; RANK, receptor activator of nuclear factor kappa B.

In summary, two characteristics of PFBC initiation have been identified: Pi imbalance (*SLC20A2* and *XPR1*) and NVU dysfunction (*PDGFRB*, *PDGFB*, *MYORG*, and *JAM2*). Cerebral Pi dyshomeostasis seems to be a common cause of PFBC. Mutations in *SLC20A2* and *XPR1* directly impair the Pi transport capacity of cerebral cells. Mutations in *PDGFRB*, *PDGFB*, *MYORG*, and *JAM2* impair the integrity of the BBB and/or BCB, leading to blood Pi leakage into the brain (Figs. 4, 5).

## The Basis of Calcification in PFBC

The above studies on the genetic etiology of PFBC revealed that it originally results from a Pi imbalance and NVU dysfunction in the brain, but the further mechanisms by which these defects lead to brain calcification remain unclear. Here, we further analyze the possible explanations by summarizing the composition of calcification in PFBC, which may also provide some potential biological markers and therapeutic targets for PFBC.

Early pathological examination revealed that the main component of brain calcification in PFBC patients is hydroxyapatite (Ca_10_[PO_4_]_6_[OH]_2_) [15, 16]. In addition, trace metals such as Al, Ar, Co, Cu, Mo, Fe, Pb, Mn, Mg, Ag, and Zn have also been detected in the calcium deposits in PFBC patients [16, 118, 119]. In 1987, Kobayashi *et al*. found that the calcium deposits were composed of a mixture of calcium salts, iron, glycoproteins, and mucopolysaccharides in one PFBC patient [17]. In 2005, Miklossy *et al*. identified the elements of calcium deposits *via* combined scanning electron microscopy and X-ray spectrometry, revealing a high level of Ca (19.97%) and P (10.08%), indicative of hydroxyapatite; C (18.11%) and O (50.49%), indicative of organic substances; and a small amount of Na (0.51%), K (0.24%), S (0.20%), and Mg (0.39%) [18]. In 2018, Jensen *et al*. analyzed calcium deposits in *Slc20a2*^−/−^ mice and found increased contents of Ca, P, Fe, Zn, Al, Mg, S, O, and N compared with the levels in non-calcified areas [120]. These studies indicate that calcium deposits in PFBC patients include a mixture of inorganic and organic substances (Fig. 4).

### Anomalous Deposition of Inorganic Substances

The inorganic substances in brain calcification are mainly hydroxyapatite, as well as low-level incorporation of metal elements, including Cu^2+^, Zn^2+^, Fe^2+^, and Mg^2+^. Intriguingly, constituent analysis of CSF from three PFBC patients detected increased concentrations of Cu^2+^, Zn^2+^, Fe^2+^, and Mg^2+^, with Ca^2+^, Na^+^, and Cl^−^ levels remaining unchanged [40, 121]. Although increased Pi levels have been shown to be a major driving factor of PFBC pathogenesis, Ca^2+^ seems to be a passive component in hydroxyapatite formation. Increased levels of Cu^2+^, Zn^2+^, Fe^2+^, and Mg^2+^ in CSF are also commonly found in some other neurodegenerative diseases such as amyotrophic lateral sclerosis, Alzheimer’s disease (AD), and Parkinson’s disease [122–124]. Whether and how an abnormality of metal minerals is engaged in the onset and/or progression of these diseases is an interesting question. Cu^2+^ and Zn^2+^ are potentially associated with amyloid beta protein (Aβ) 42 plaque formation, oxidative stress, and neurodegeneration [124, 125]. Similarly, Fe^2+^ has been suggested to be involved in the release of oxidative factors and inflammation leading to neurodegeneration [126–128]. In humans, plasma Cu^2+^, Zn^2+^, and Fe^2+^ concentrations are much higher than those in CSF [124]. Disrupted BBB integrity is also associated with increased levels of Cu^2+^, Zn^2+^, and Fe^2+^ in the brains of AD patients [129]. Thus, combined with studies showing BBB impairment in a PFBC patient and *Pdgfrb/Pdgfb*-deficient mice [18, 89, 91], increased levels of Cu^2+^, Zn^2+^, and Fe^2+^ in PFBC patients might be associated with increased BBB permeability leading to blood leakage. Interestingly, amyloid precursor protein (APP) and amyloid precursor-like protein 2 (APLP2), which harbor binding sites for Cu^2+^ and Zn^2+^ [130], accumulate in calcified nodules [131]. This feature may contribute to Cu^2+^ and Zn^2+^ deposition in calcifications.

### Anomalous Changes in Organic Substances

Many studies have shown that ectopic calcification is not a simple passive process but a complex and highly-regulated active process involving the activation of cell signaling pathways and the production of calcification inhibitors and hormone regulation [132, 133], some of which may be detected in the organic components of calcium deposits. Few early reports described organic substances in brain calcifications, and systematic studies are particularly rare. Recently, Nahar *et al*., using liquid chromatography with tandem mass spectrometry, identified the protein components in brain calcifications in *Pdgfb*^ret/ret^ mice, and found 10 proteins exclusively in calcified nodules, including three bone-formation inhibitors, fetuin A (alpha 2-Heremans–Schmid glycoprotein, AHSG), matrix gla protein (MGP), and OPN [134–136]; an ancestral mineralization protein sparc-like 1 (SPARCL1) [137]; three neuronal function-associated proteins APP, APLP2, and the neurosecretory protein nerve growth factor inducible (VGF) [138, 139]; two hormonal activity-associated proteins secretogranin-1 (CHGB) and chromogranin A (CHGA) [140]; and the lysosomal proteinase cathepsin Z (CTSZ) [141]. Immunohistochemical staining has confirmed the presence of OPN, APP, APLP2, SPARCL1, VGF, and CHGA in calcium deposits in *Pdgfb*^ret/ret^ mice, and similarly, APP, APLP2, and SPARCL1 have also been found in calcium deposits in *Slc20a2*^−/−^ mice [131].

Among the organic content in calcium deposits, four proteins (AHSG, MGP, OPN, and SPARCL1) are well-known to modulate bone formation and homeostasis [136, 142–144], implying that brain calcification undergoes similar pathological processes. In addition, Zarb *et al*. analyzed the cellular circumstances of calcified nodules in *Pdgfb*^ret/ret^ mice and showed the presence of bone matrix proteins (collagen I, osteocalcin, and OPN) in calcification deposits. Moreover, the cells around calcium deposits were found to express markers of osteoblasts (RUNX2), osteoclasts (cathepsin K and RANK), and osteocytes (sclerostin). Consistent with this, osteogenic niches surrounding brain calcium deposits have also been reported in 3 PFBC patients carrying mutations in *PDGFRB* (p.Arg695Cys), *SLC20A2* (p.Met1_Val652del), or *SLC20A2* (p.Ser113*) [145]. In addition, accumulated reactive astrocytes and microglia have been observed around calcium deposits in one PFBC case and *Pdgfb*^ret/ret^ mice [18, 131, 145]. Reactive astrocytes abnormally express the bone matrix protein OPN and the osteocyte marker podoplanin accompanied by a neurotoxic response and oxidative damage [145, 146], indicating that reactive astrocytes play an important role in the formation of an osteogenic environment but remain difficult to identify as promoters or inhibitors of brain calcification. Reactive microglia abnormally express the osteoclast markers cathepsin K and RANK and have been demonstrated to be beneficial for pathological brain calcification; their deficiency intensifies brain calcification *via* triggering receptors expressed on myeloid cells 2 (TREM2) [146]. Considering that VSMCs can be induced by high Pi to undergo differentiation to osteoblast-like cells and form calcification [58, 59], brain calcification associated with osteogenic environments and vessels suggests that ossification of some cerebral cells induced by increased Pi levels in the brain might be a common process in PFBC.

The proteins associated with neuronal function APP, APLP2, and VGF are associated with AD, especially APP, which is key to the formation of Aβ plaques in AD [147–149]. However, these proteins deposited in calcified areas lack a β-pleated sheet conformation and structural regularity [146], in contrast to Aβ plaques in patients with AD. Recently, APP and APLP2 messenger RNAs have been shown to be highly expressed in bone [150]. APP and Aβ (a small proteolytic fragment of APP) have also been identified in the bone, largely in osteocytes and the bone matrix. Furthermore, Aβ enhances the differentiation and activation of osteoclasts [151, 152]. Thus, the accumulation of APP and APLP2 in brain calcification seems to be caused by a mechanism similar to that of AHSG, MGP, and OPN accumulation, contributing to the formation of osteogenic environments. Another protein component of calcium deposits, CTSZ, a lysosomal proteinase cathepsin, is produced in both osteoclast and osteoblast lineages [153–155], suggesting that CTSZ in calcification areas also contributes to the formation of osteogenic environments. Nahar *et al*. hypothesized that VGF and the hormonal activity-associated proteins CHGB and CHGA passively adhere to calcifications because the expression of these proteins is mostly restricted to neurons [131]. However, more experiments are needed to identify the key proteins because ectopic calcification is highly regulated by hormones.

In summary, analysis of the composition of calcification in PFBC reveals that brain calcification formation is a complex process involving various inorganic and organic substances. The discovery of osteogenic environments in the calcified brain suggests that cell ossification induced by increased Pi levels in the brain seems to be a common process of calcification in PFBC (Fig. 4).

## Prevention and Treatment of PFBC

Although PFBC has been studied for >170 years, no specific prevention or treatment has been discovered. The identification of causative genes (*SLC20A2*, *PDGFRB*, *PDGFB*, *XPR1*, *MYORG*, and *JAM2*) and studies of its pathogenesis may provide some potential therapeutic targets for the prevention and treatment of PFBC.

### Treatment Cases

Currently, the clinical treatment of PFBC patients is mainly symptomatic-oriented. Due to the large clinical heterogeneity of PFBC symptoms, different medications are used, including antipsychotics, anticonvulsants, antidepressants, mood stabilizers, antiparkinsonism-directed and anti-incontinence-directed medications, analgesics, and benzodiazepines [156]. However, only a few patients have shown improved control of their symptoms (e.g., Tololeski *et al*. reported an adolescent patient for whom quetiapine treatment successfully attenuated acute psychosis, and Uno *et al*. reported a middle-aged patient who was relieved of recurrent psychosis with risperidone) and no positive effect on brain calcification has been shown; moreover, the risk of symptom relapse is high [26, 157].

Effective therapies to control the progress of brain calcification have rarely been reported. Treatment with nimodipine, a calcium channel blocker in the CNS, has been unsuccessful in attenuating PFBC [10]. Bisphosphonates, with a structure analogous to that of pyrophosphoric acid, are mainly used in the clinical treatment of osteoporosis, and they can bind to hydroxyapatite and preferentially localize to the site of active bone remodeling, reducing the process of bone resorption by inhibiting osteoclast activity [158]. In a small number of PFBC patients, oral bisphosphonate administration has been reported to improve symptoms, but brain calcification was not reduced, as indicated by CT imaging. Loeb described a middle-aged patient treated with disodium etidronate, a bisphosphonate, which produced a twofold improvement in his speech and gait rate but without affecting spasticity, dystonia, ataxia, or brain calcification [159]. In addition, Loeb *et al*. reported that two patients treated with disodium etidronate presented with a significant reduction in seizure frequency and headaches but no reduction in calcium deposit size, as determined by CT imaging [160]. Recently, Oliveira *et al*. reported 7 patients treated with alendronate, another bisphosphonate, and reported improvements and stability without obvious side-effects in some of these patients, particularly younger patients, but no specific change in brain calcification was identified by CT imaging [161]. Although the effectiveness of bisphosphonates for PFBC needs to be confirmed with more evidence, they may be potentially effective drug options for the nonspecific treatment of PFBC conditions.

### Speculation on Potential Prevention and Treatment

Hypovitaminosis D was reported in a PFBC patient carrying an *SLC20A2* mutation [162], and vitamin D receptor (VDR) knockout in mice resulted in symmetrical thalamic calcification [163], suggesting that deficient vitamin D or its receptor is associated with brain calcification. Furthermore, *SLC20A2* expression is positively regulated by vitamin D, which reduces calcification *in vitro* [164]. Thus, vitamin D may be a potential treatment for PFBC patients, but the detrimental effects of high levels of vitamin D should be noted.

In general, the frequency of nonsense variants in *SLC20A2*, *PDGFRB*, *PDGFB*, *XPR1*, *MYORG*, and *JAM2* reaches 13.2% [9]. Considering that PFBC patients carry these variants, Peters *et al*. suggested that ataluren, an agent with the potential for treating a broad range of genetic diseases caused by nonsense variants [165], might be a potential option [166].

PDGFR-β is a tyrosine kinase receptor, and its activation further activates many downstream signaling pathways. Considering this, Lemos *et al*. speculated that PDGFRB might be an interesting therapeutic target for PFBC patients with PDGFB or PDGFRB mutations and that drugs modulating the PDGF-B/PDGFR-β signaling pathways might be potential treatments [156]. However, animal experiments and case studies to confirm this possibility are lacking, and the mechanism by which the downstream pathways affected by PDGF-B/PDGFR-β impairment cause calcification also needs further study.

Analyses of the genetic etiology of PFBC have prompted the hypothesis that cerebral Pi dyshomeostasis may be a common cause of PFBC. In addition, in *SLC20A2*-deficient brains of both humans and mice, significant elevation of CSF Pi levels has been detected and is increasingly accepted as a marker for PFBC [40, 42, 53]. Thus, we inferred that a high level of brain Pi might be a therapeutic target for PFBC prevention. Reducing Pi intake through reasonable dietary intervention or restoring the normal Pi transport by supplementation of PiT2 expression with viral vectors and gene editing technology in the brain may be promising research directions.

Cell ossification occurs in PFBC and seems to be a common process. Reactive microglia have been shown to be beneficial for inhibiting pathological brain calcification, possibly through matrix degradation mediated by the TREM2-dependent protein cathepsin K (Fig. 4). Hence, inhibiting cell ossification and increasing bone resorption-promoting protein release might be interesting lines of research for exploring PFBC treatment.

Here, we have summarized the current research progress of prevention and treatment for PFBC in two aspects, reported treatment cases and potential prevention and treatment.

## Conclusions and Perspectives

In conclusion, the studies analyzed three aspects of PFBC research mainly based on functional research on the reported causative genes in the past decade. First, two characteristics of the genetic etiology of PFBC are classified: Pi imbalance and NVU dysfunction in the brain; in addition, cerebral Pi dyshomeostasis seems to be a common cause of PFBC that can be directly influenced by mutations in *SLC20A2* and *XPR1* and may be indirectly influenced by mutations in *PDGFRB*, *PDGFB*, *MYORG*, and *JAM2*. Second, based on studies of the composition of calcified nodules, cell ossification induced by increased Pi levels in the brain seems to be a common process of calcification in PFBC. Third, the current research status of PFBC treatment is summarized as follows: no specific prevention or treatment is available, and we highlight several potential prevention/treatment options and therapeutic targets: (1) bisphosphonates target calcification and may be effective against some PFBC symptoms; (2) vitamin D may be effective for PFBC patients with *SLC20A2* mutations and hypovitaminosis D because it increases *SLC20A2* expression; (3) ataluren is an agent with the potential for suppressing nonsense mutations and may be effective for PFBC patients with nonsense mutations; (4) as an interesting therapeutic against PDGFR-β, drugs modulating PDGF-B/PDGFR-β signaling may be potential treatments for PFBC patients with PDGFB or PDGFRB mutations; (5) as a therapeutic target to re-establish cerebral Pi homeostasis, reducing Pi intake or restoring the normal Pi transport in the brain may be interesting research directions; and (6) inhibiting the process of cerebral cell ossification may be an interesting therapeutic option to inhibit calcification in PFBC (Fig. 5).

To effectively control the progress of brain calcification and accompanying brain insults, more studies are needed. First, the genetic basis in ~40% of PFBC patients remains unclear [9, 26–39, 167]; the application of novel genetic sequencing technology is critical to identify new causative genes to expand the genetic spectrum of PFBC. Second, dissection of the cellular and molecular mechanisms underlying brain calcification requires inter-disciplinary collaborative efforts to establish a systematic understanding, especially of the signaling network of PFBC-associated genes and the osteogenic hypothesis. Third, in addition to mouse models of PFBC, novel animal models, especially large animal models (e.g., rat and monkey), are needed for the simulation of PFBC pathogenesis and translational research. Fourth, more studies on the mechanism of action of potential drugs and the identification of the targets of potential drugs will help apply the treatment of monogenic PFBC to the treatment of common brain calcification treatments.
